# A New Biotin Labeling and High-Molecular-Weight RNA Northern Method and Its Application in Viral RNA Detection

**DOI:** 10.3390/v14122664

**Published:** 2022-11-28

**Authors:** Xi Zhang, Qingling Zhang, Long Cheng, Dan Liu, Hongzheng Wang, Yingjia Zhou, Liqun Ma, Jubin Wang, Feng Li

**Affiliations:** Key Laboratory of Horticultural Plant Biology (MOE), College of Horticulture and Forestry Sciences, Huazhong Agricultural University, Wuhan 430070, China

**Keywords:** virus, high-molecular-weight RNA, Northern blot, biotin-labeled probe, denaturing agarose gel

## Abstract

Viruses cause severe crop losses. Studying the interaction between viruses and plants is very important for development of control measures. Northern blot is a well-accepted but very challenging technique to monitor the infection of viruses. Here, we modified the high-molecular-weight (hmw)RNA Northern blot experiment process, utilizing vertical electrophoresis to separate the RNA with denatured agarose gel. This protocol is compatible with regular equipment for Western blots and small RNA Northern blots and requires less input of total RNA. A new method to label the probe with biotin was also developed, which requires commonly used T4 DNA polymerase and detects viral RNA with high sensitivity. These new protocols made hmwRNA Northern blot cost-effective and easy-to-operate, very suitable for studying virus–host interactions.

## 1. Introduction

Viruses impose serious threats on plants and agriculture and virus research is important for developing counter-measures for various devastating viral pathogens. Virus detection forms the foundation for virus research and many methods have been developed to quantify virus levels in plants [1]. Traditional methods such as bioassay on indicator plants are cumbersome and time-consuming. Protein-based detection methods, such as enzyme-linked immunosorbent assay (ELISA), are sensitive and fast but rely on virus-specific antibodies, which are not always available. Molecular detection methods require virus sequence information and synthesis of molecular probes and include mainly amplification-based methods and hybridization-based methods [1]. Owing to the development of high-throughput sequencing [2] and maturation of oligo DNA synthesis techniques, molecular detection methods have become everyday techniques for a virus research lab. Amplification-based methods include reverse transcription followed by various amplification methods, such as polymerase chain reaction (qRT-PCR), loop-mediated isothermal amplification (LAMP), Recombinase polymerase amplification (RPA), and LAMP and RPA combined with clustered regularly short palindromic repeat-associated systems (CRISPR/Cas), which have the advantage of a fast process, high sensitivity, and low labor intensity [3]. However, their advantages also bring some risks. RNA quality and RT efficiency will lead to deviation due to high sensitivity, and the RNA size is not clear. Northern blot is also a very commonly used hybridization-based method for detecting various RNAs including viral RNAs, and its results can directly reflect transcript abundance and size [4,5]. In traditional Northern blot, RNA is fractionated using agarose gel electrophoresis in a horizontal gel apparatus and transferred onto a nylon membrane using a vacuum transfer unit or using the paper blotting method, which requires a large amount of blotting paper or transfer buffer, and thus is not environmentally friendly [4,6].

Although Northern blot is considered a gold-standard for detecting RNA accumulation levels, limitations such as laborious procedure, requirements of radioactive probes, and low sensitivity warrant continuous efforts to improve this technique. Zhao and colleague showed that pre-staining RNA with ethidium bromide (EtBr) had a negative impact on hybridization efficiency [7]. For enhanced detection sensitivity, Ahmad et al. modified the existing hybridization protocol of Northern blot and conducted liquid hybridization in a final volume of 25 μL through stepwise annealing, and then excess probes were removed by enzymatic digestion before gel electrophoresis and membrane transfer [8]. In a conventional Northern blot experiment, the hybridization usually take place in around 5–10 mL hybridization buffer after RNA transfer and is crosslinked to the membrane. This modification increases probe concentration greatly by reducing the hybridization volume, thus obtaining higher sensitivity, but it loses size information of the target RNA. More recent studies have shown that using moderate washing conditions after hybridization can enhance the capacity of RNA blots for repeated hybridization to up to eight rounds on a single blot [9].

Radioactive probes have been used since the Northern blot system was established, despite their high detection sensitivity and simplicity of operation, high cost, safety problems, restriction rules, and short shelf life, necessitating efforts in developing nonradioactive probes to overcome these shortcomings. As a result, digoxigenin (DIG)- [10] and biotin-based probe labeling methods were developed for both mRNA and sRNA detection. DIG and biotin can be incorporated into probes during probe oligo (or PCR primer) synthesis through chemical reaction or during enzymatic synthesis from a DNA template by adding a DIG- or biotin-modified nucleotide [8,11,12]. DIG- and biotin-labeled probes are then detected by anti-DIG antibodies and streptavidin, respectively, coupled to alkaline phosphatase (AP) or horseradish peroxidase (HRP) [12,13]. Recently, near-infrared fluorescent dye-labeled probes were reported for Northern probe labeling, and require special equipment to detect the signal [13].

In the study of plant–virus interaction, small interfering (si)RNA is an important player in mediating plant resistance against viruses. Plant Dicer-like 4 and 2 cleave viral double strand (ds)RNA into 21 and 22 nt siRNAs, which are incorporated into Argonaute 1 and 2 proteins to form RNA-induced silencing complex (RISC) with only one strand retained. The viral siRNA programs RISC target viral RNAs for degradation or translational repression based on sequence complementarity [14]. Thus, analysis of viral siRNA is important in studying plant–virus interaction. Although viral genomic RNA and viral siRNA are both RNA in chemical composition, viral siRNA Northern blot requires different equipment sets due to their size difference. For siRNA Northern blot, the RNA sample is separated by polyacrylamide gel in a vertical gel system and transferred by semidry transfer system [15]. The probe for siRNA detection is also prepared differently from that for viral genomic RNA [16]. These different requirements inevitably increase the cost of experiments and make related equipment not cost-effective.

To further improve the Northern blot procedure, we modified both probe labeling and the gel electrophoresis protocol to make the procedure simpler and the equipment compatible with other experiments, thus potentially reducing equipment costs. In this study, a hairpin probe structure was developed and optimized, and was extended by T4 DNA polymerase such that multiple biotin-labeled dCTP was incorporated. The conventional horizontal gel electrophoresis was replaced with a thin vertical gel electrophoresis using regular protein or a small RNA gel electrophoresis system. Semidry gel transfer was conducted to transfer RNA from gel to the membrane, which was also the same for small RNA and protein transfer. We applied these modified procedures for detection of several plant viruses in plant samples and obtained high sensitivity of viral RNA detection. These improvements will be of general interest for virologists in viral-infection analysis.

## 2. Materials and Methods

### 2.1. RNA Isolation and Sample Denature

Total RNA was isolated from leaves using Transzol (Transgen, Beijing, China) according to the manufacturer’s instructions and dissolved in DEPC water. TMV- and ToMV-infected samples were taken from wide-type tomato plants infected by each virus. TRV- and CMV-infected samples were harvested from *Nicotiana benthamiana* leaves 7 days after agroinfiltration. RNA samples of required amounts were transferred into new 1.5 mL microcentrifuge tubes and vacuum-dried using a vacuum concentrator (Eppendorf, Hamburg, Germany). Then, RNA pellets were dissolved in 20 μL 50% formamide (Sigma, Saint Louis, MO, USA), heated at 65 °C for 10 min, and put on ice for 2 min. Before loading the RNA sample into the gel, 4 μL RNA loading buffer (0.25% (*w*/*v*) Bromophenol Blue, 0.25% (*w*/*v*) Xylene cyanol FF, 40% (*w*/*v*) Sucrose in water) was added.

### 2.2. Probe Preparation

PAGE-purified Oligo was ordered from a commercial provider and diluted to 10 μM before use (Table S1). To assemble the labeling reaction, 1 μL probe oligo (usually, we utilize two probes for one virus detection), 1 μL dATP of 1 mM (NEB, Ipswich, MA, USA), 1 μL biotin-16-dCTP of 1 mM (Jena Bioscience, Jena, Thuringia, Germany), 1 μL T4 DNA polymerase (NEB, Ipswich, MA, USA), 1 μL 10× T4 DNA polymerase buffer (NEB, Ipswich, MA, USA), and DEPC water were added to make a 10 μL reaction. The reaction was incubated at 25 °C for 15 min and then at 37 °C for 1 h. The entire reaction was added to the hybridization tube during the hybridization step. For terminal deoxynucleotidyl transferase reaction, 1 μL 10× TdT buffer (NEB, Ipswich, MA, USA), 1 μL CoCl_2_ of 2.5 mM (NEB, Ipswich, MA, USA), 1 μL probe oligo, 1 μL biotin-16-dCTP of 1 mM (Jena Bioscience, Jena, Thuringia, Germany), 1 μL Terminal nucleotide Transferase (NEB, Ipswich, MA, USA), and DEPC water were added to make a 10 μL reaction. The reaction was incubated at 37 °C for 1 h.

### 2.3. Gel Running, Transferring, and Staining

To make the formaldehyde denaturing agarose gel, 0.6 g agarose, 5 mL 10× MOPOS buffer was added into 36 mL RNAse free ddH_2_O and boiled in the microwave until it completely dissolved. The boiled gel was cooled down to 60 °C, 9 mL 37% formaldehyde was added, and it was poured into the gel cassette and a comb was placed. After the gel was solidified, the comb was taken off carefully because the agarose gum hole spacers are fragile. Gel electrophoresis was performed with vertical electrophoresis equipment (JunYI, Beijing, China) using 1× MOPS buffer at 120 V for various lengths of time. After electrophoresis, glass plates were removed with care from the glass plate–agarose gel–glass plate sandwich using a plastic plate separation tool. The agarose gel was gently placed in the semidry transfer unit (JunYi, Beijing, China) or Trans-Blot Turbo (Bio-Rad, Hercules, CA, USA) to transfer RNA onto the Amersham Hybond-N+ nylon membrane (Cytiva, Buckinghamshire, UK). Electric current of 1 mA per cm^2^ of membrane was used during transferring process. Different lengths of time were tested. The agarose gel was stained in EtBr solution for 10 min and briefly washed in ddH_2_O before taking a picture using the Molecular Imager Gel Doc EX System (NEWBIO Industry, Tianjin, China).

### 2.4. Crosslink and Hybridization

The membrane was crosslinked using CL-1000 Ultraviolet Crosslinker (UVP, Upland, CA, USA) with the setting of 1200 μJ and treated twice. Then, the membrane was stained in the methylene blue buffer (0.04% (*w*/*v*) Methylene blue, 0.5 M Sodium acetate, pH 5.2) for 10 min in a plastic box and washed in ddH_2_O five times, approximately 3 min each time, before taking a picture. Membrane pre-hybridization was conducted using commercial RNA hybridization buffer (Sigma, Saint Louis, MO, USA) for 20 min at 37 °C, then the labeled probe was added into the hybridization tube and incubated at 37 °C for more than 8 h.

### 2.5. Chemiluminescence Detection and Stripping the Probes

After hybridization, the membrane was treated using the Chemiluminescent biotin-labeled nucleic acid detection kit (Beyotime, Shanghai, China) according to the manufacturer’s instruction immediately before imaging using a ChemiDoc XRS+ system (BioRad, Hercules, CA, USA), with different exposure times according to the signal intensity. Before re-probing the membrane, the probe was washed from the membrane using stripping buffer (2% SDS, 10 mM Tris, pH 7.4) at 68 °C two times, for 20 min each time.

## 3. Results

### 3.1. Design and Optimization of Probe Structure

**Figure 1:**
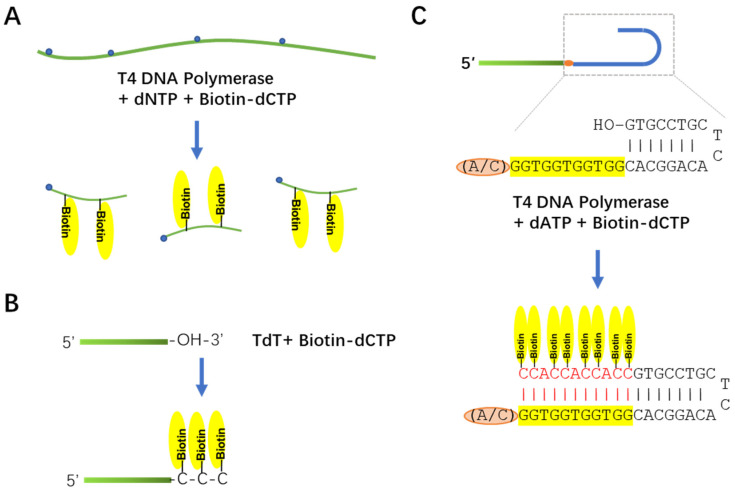
Mechanism of probe labeling. (**A**) Mechanism of random priming labeling. Green flexible line represents double-stranded DNA template while the shorter and thinner green line represents single-stranded DNA probe synthesized in labeling reaction. Blue dots represent random hexamer primers. Yellow ovals represent biotin module attached to cytosine. (**B**) Labeling of short DNA oligo using terminal nucleotide transferase. Green bars represent probe sequences. TdT, terminal deoxynucleotidyl transferase. (**C**) Short hairpin enabled T4 DNA polymerase-mediated labeling method. Green bar, probe sequences; brown oval, insolating nucleotide (A/C); blue “U” shape line, hairpin structure.

A biotin-labeled probe is a safe and sensitive reagent for molecular detection. Currently, probes for high-molecular-weight (hmw)RNA Northern blot are usually made by the random primer mechanism, producing long single-strand DNA probes with biotin-labeled nucleotide [17,18] (Figure 1A). Probes for small RNA Northern blot are about 20–30 nt long and usually labeled by chemical synthesis or terminal nucleotide transferase reaction [19] (Figure 1B). T4 DNA polymerase is a commonly used lab reagent and has broad application in everyday lab work. We proposed a new way to label short probe sequences with T4 DNA polymerase (Figure 1C). In this method, a hairpin sequence and a single-strand GT-rich sequence were attached to the 3′ end of a given probe sequence. The 3′ end of the hairpin served as a primer for T4 DNA polymerase. Only dATP and biotin-dCTP were added, so that the polymerization reaction would stop at the A or C nucleotide inserted between the probe sequence and the GT-rich sequences (Figure 1C).

**Figure 2:**
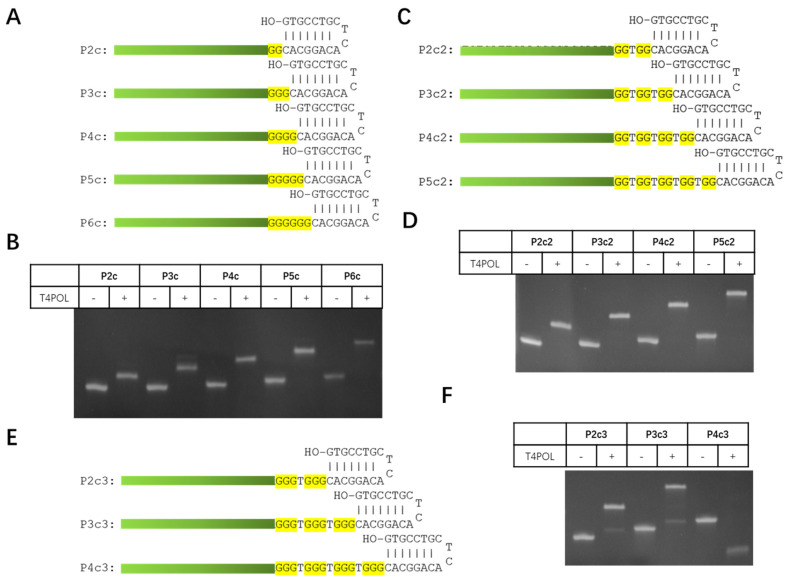
Effect of G repeats on probe labeling efficiency. (**A**) Sequences and structures of probe with two to six G repeats in template strand. (**B**) PAGE analysis of unlabeled and labeled P2c–P6c probe DNA. T4POL, Polymerization reaction of 15 μL with T4 DNA Polymerase. (**C**) Sequences and structures of probe with two to five GG repeats separated by T in template strand. (**D**) PAGE analysis of unlabeled and labeled P2c2–P5c2 probe DNA. (**E**) Sequences and structures of the probe with two to three GGG repeats separated by T in template strand. (**F**) PAGE analysis of unlabeled and labeled P2c3–P4c3 probe DNA. Probe IDs are indicated to the left. Letters in yellow background indicate template nucleotides for T4 DP.

To test the proposed probe labeling method, three sets of hairpin oligoes were designed: in the first set oligoes, a probe sequence was attached to a set of hairpins with 5′ overhang of 2 to 6 G (Figure 2A); in the second set oligoes, a probe sequence was attached to a set of hairpins with 5′ overhang of 2 to 5 GG dinucleotide spaced by a T (Figure 2C); in the third set oligoes, a probe sequence was attached to a set of 5′ overhangs of 2 to 4 GGG triple nucleotides spaced by a T (Figure 2E). These different structures were designed to test the efficiency of probe labeling, because of potential interference in the polymerase reaction by the biotin modification in dCTP. All these hairpin oligoes were diluted into 10 μM and 1 μL of each was labeled through T4 DNA Polymerase reaction (see Section 2.2. PAGE analysis of the products showed that in the presence of the T4 DNA polymerase reaction, all oligoes shifted to higher molecular-weight bands (Figure 2B,D,F), indicating that polymerase reactions were successful for all hairpin structures.

**Figure 3:**
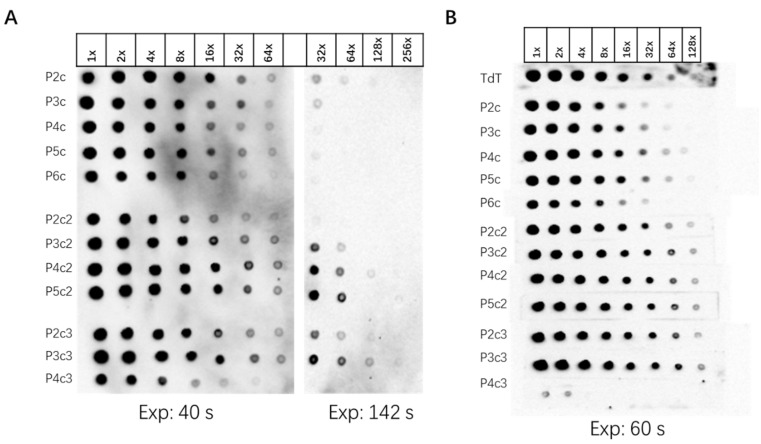
Sensitivity test of labeled probes in dot blot analysis. (**A**) Detection of labeled probes using biotin-detection kit. The labeled probe was purified with a gel filtration column, diluted as indicated on top, added onto the N+ nylon membrane, and UV-crosslinked. Here, 1× represents the 10 pmoles probe. (**B**) Detection of hybridized membrane using biotin-detection kit. Oligo complementary to probe sequences was diluted as indicated on top, added onto the N+ nylon membrane stripe, UV-crosslinked, and hybridized with each probe (indicated to the left) individually. TdT, a TdT-labeled probe used as a control. Here, 1× represents 10 pmoles oligo. Exp, exposure time.

The sensitivity of probe detection was tested In two ways. In the first way, series dilution of each probe was spotted onto one membrane, which was subjected to UV-crosslink. The membrane was then developed by biotin detection kit. The result showed that P3c2, P4c2, P5c2, P2c3, and P3c3 showed relatively stronger signals at higher dilution spots (Figure 3A). This is consistent with the higher number of biotin-dCTP incorporated, except for P4c3 which was predicted to contain the highest number of biotin-dCTP but lowest sensitivity. In the second analysis, DNA oligo complementary to the probe sequence was spotted onto a strip of N+ nylon membrane in a series dilution and cross-linked by UV irradiation (Figure 3B). Each probe labeling reaction was hybridized with a cross-linked membrane in a separate hybridization tube to test their sensitivity in detecting their complementary oligo (see Section 2.4 and Section 2.5). The results showed that a stronger signal at 64× dilution was detected with P3c2, P4c2, P5c2, P2c3, and P3c3 probes (Figure 3B). These results show that there is a correlation between the number of biotin-dCTP incorporated, but it is not a linear relationship. The probe with five or fewer biotin-dCTP incorporated, including the TdT-labeled probe, showed similar and weaker signals compared to that with six or more biotin-dCTP, except P6c and P4c3.

### 3.2. Optimization of Gel Running and Transferring Procedure

**Figure 4:**
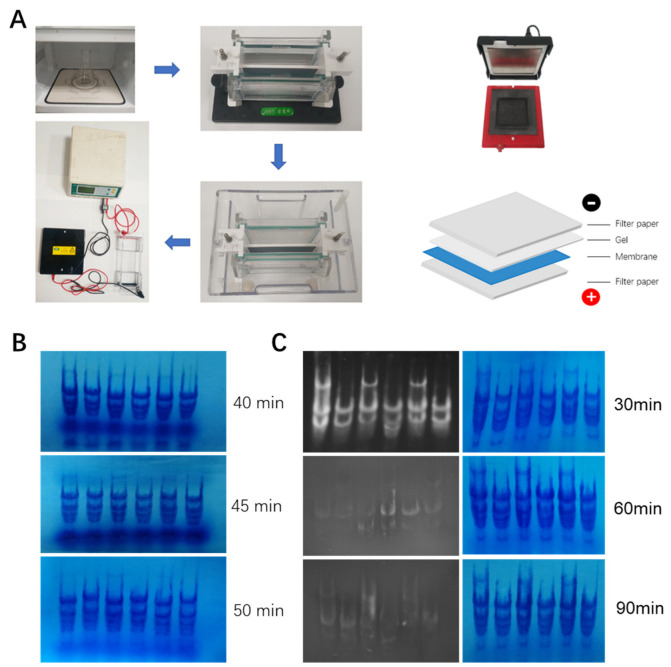
Optimization of gel running and transferring condition. (**A**) Pipeline of new gel running and transferring procedure. (**B**) Bromophenoblue staining of membrane transferred from gels running for different time indicated to the right. A 20 μg total RNA sample was loaded in each lane. Gel was transferred for 90 min. (**C**) Bromophenoblue staining of membranes after transferring for indicated time (in the right). A 20 μg total RNA sample was loaded in each lane. Gel was run for 45 min.

To circumvent the problem of traditional methods for hmwRNA Northern analysis, we adopted the vertical gel electrophoresis system and semidry transfer system for hmwRNA Northern blot, which are commonly used for small RNA Northern blot and Western blot (Figure 4A). Usually, polyacrylamide gel is used for small RNA and protein separation in vertical systems, while for hmwRNA, agarose gel was required. A 1.2% agarose gel of 1.5 mm thick, 8 cm high, and 8 cm wide was made using the gel casting set of the vertical gel system. Total RNA sample of 20 μg from *Nicotiana benthamiana* leaves was prepared, denatured, and loaded into the vertical gel system (see Section 2.1).

Gel systems were run at 120 V constant voltage for 40, 45, and 50 min to optimize the running time. Then, the gel cassette was gently disassembled, and the agarose gel was carefully transferred to semidry gel transfer system to avoid gel breaking. Gel transfer was conducted for 90 min and then membrane was briefly rinsed in DEPC-treated water and stained (see Section 2.3 and Section 2.4). The staining results showed that running gel for 45 min was enough to clearly separate ribosomal RNA into discrete bands (Figure 4B). To further optimize the gel transfer process, three gels were run in parallel for 45 min and transferred for 30, 60, and 90 min. When gel transfer was completed, both agarose gel and membrane were stained (see Section 2.3 and Section 2.4) and imaged. As seen in Figure 4C, there was still strong ribosomal RNA staining in the gel after 30 min transferring. In contrast, there was faint staining in the gels that were subjected to 60- or 90-min transfer processes. All three membranes showed well-separated strong ribosomal RNA staining.

These results showed that a vertical gel system and semidry transfer system could be adopted for hmwRNA Northern blot analysis. For the equipment we used, 45 min gel running and 90 min gel transferring could reach an optimal result.

### 3.3. Virus Detection Using Newly Optimized Methods

**Figure 5:**
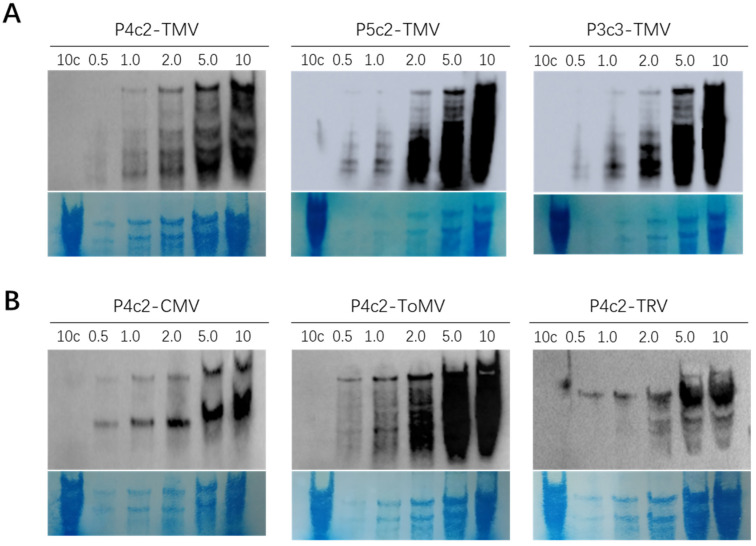
Detection of different viral hmwRNAs using new Northern blot protocol. (**A**) TMV blots hybridized using probes attached to different G repeats. (**B**), CMV-, ToMV-, and TRV-infected samples were analyzed with probes indicated above each blot. The first lane in each blot was loaded with a 10 μg total RNA sample from uninfected samples, indicated as 10c; in all other lanes, the same virus-infected sample was loaded with the amount indicated on top of each lane in μg.

Next, we set to apply the above optimized methods in detection of various viruses. In order to test the sensitivity of the method, various amounts of total RNA samples were prepared, which were extracted from TMV-, CMV-, ToMV-, or TRV-infected plants ranging from 0.5 μg to 10 μg. A non-infected total RNA sample of 10 μg was used as negative control. These RNA samples were separated by the vertical gel system and transferred to N+ nylon membrane using semidry transfer system using optimized settings. After crosslinking and staining, the ribosomal RNA bands on the membrane showed gradient of blue staining as expected on each membrane (Figure 5A,B, bottom).

Three TMV RNA membranes were hybridized with a 40 nt probe sequence attached to three different G repeat templates, P4c2-TMV, P5c2-TMV, and P3c3-TMV, that showed higher sensitivity in dot blot analysis. The results show that all probes detected a specific and clear signal at 1.0 μg TMV-infected sample corresponding to the TMV genomic RNA. A very faint TMV gRNA band was detected by the P5c2-TMV at 0.5 μg (Figure 5A, top). The blots of CMV-, ToMV-, and TRV-infected RNAs were hybridized with a 40 nt probe sequence complementary to each virus attached to four GG repeats, P4c2-CMV, P4c2-ToMV, and P4c2-TRV, which represent the lower end of the detection sensitivity among the three types of G repeats. The results showed that the clear genomic RNA3 band and subgenomic RNA4 band were clearly detected in a 0.5 μg CMV-infected sample. Similar sensitivity for detection of the ToMV and TRV RNA2 genomic RNA band was also achieved (Figure 5B). These results showed that the new Northern blot procedure was suitable for viral RNA analysis with high sensitivity.

## 4. Discussion

For a molecular virology laboratory, Northern blot detection of viral genomic RNA, subgenomic RNA, or siRNAs is frequently conducted. Traditional hmwRNA Northern blot is conducted with a horizontal gel electrophoresis system. Although the horizontal gel system is regularly used in laboratories for DNA analysis, to avoid degradation by RNase that often presents in DNA preparation, additional horizontal gel electrophoresis system is usually required for RNA Northern blot. For the siRNA gel, running a vertical gel system is required due to high-resolution requirement. The equipment for hmwRNA gel transfer is also different from that of siRNA gel. Thus, two separate sets of equipment are required for analyzing viral genomic RNA and siRNA, which increases lab investments and effort in training newcomers. In this study, we optimized the procedure to use a vertical gel system and semidry transfer system for hmwRNA Northern blot, which are usually used for siRNA Northern blot. Combined with the newly established T4 DNA polymerase-mediated probe labeling using biotin-dCTP, sensitive detection of viral hmwRNA was achieved. These optimizations in gel running and transferring and new probe labeling would make viral hmwRNA and sRNA Northern blot easier and more cost-effective. In traditional hmwRNA Northern blot, the agarose gel is thick enough to keep its integrity during handling, while in the new procedure the agarose gel is very thin and fragile. Thus, special care need be taken to handle the thin agarose gel during vertical gel electrophoresis and semidry transfer. Some tricks could help to keep the gel integrity during handling, for example, applying water repellent, such as Rain-X, onto the glass plates before loading agarose gel and using a plate separator to open the glass. Special care is also needed when pulling out the comb from the plates because the teeth of the gel are easily broken during this process. For a better comparison between the new protocol developed in this study and the traditional Northern blot protocol, we summarize the time frame, major differences, and cautions for the major steps in each protocol (Table 1).

For sRNA and hmwRNA Northern probe labeling, T4 PNK-mediated phosphorylation of short DNA oligo using [γ-^32^P]ATP and random priming using [α-^32^P]dCTP were frequently used. However, due to restricted lab administration and inconvenient importing process, it is not a reliable method for daily work in most laboratories in China. Biotin- or digoxigenin-labeled probes are acceptable alternatives. Chemical synthesis of a biotin-labeled probe is expensive, especially when detecting viral siRNAs mixture of several probes is required, which execrates the cost of experiments. Terminal nucleotide transferase-mediated terminal labeling is suitable for individual DNA oligo or a mixture of DNA oligo. However, this enzyme adds only between one and three biotin-dCTP to the 3′ end of the DNA oligo due to interference by the biotin group in the base structure [19]. Furthermore, addition of multiple C in the single-strand DNA oligo will potentially increase nonspecific binding of the probe. In this work, we developed a simple method to label short oligo probe using T4 DNA polymerase, a commonly used lab enzyme. With this labeling method, we achieved sensitive detection of several different viruses in hmwRNA Northern blot experiments, confirming the effectiveness of this method. The current probe design allowed addition of up to 10 biotin-dCTP into the probe. However, the sensitivity of labeled probes with different numbers of dCTP did not show significant difference among these probes. The biotin groups were detected using HRP-streptavidin-based assay [16]. Streptavidin binds biotin as a homo tetramer and can bind up to four biotin groups, and each tetramer binds two pairs of biotin in head-to-head orientation [20]. The special binding model of streptavidin may not allow a linear increase in streptavidin molecule number with the number of biotin groups in a short rigid duplex DNA. Thus, there is still more room to optimize the structure of probe to obtain better sensitivity.

The method for immobilization of nucleic acids on nylon membrane is also an important factor influencing probe sensitivity. UV-crosslink relies on the photoinduced reaction between base group in nucleotide and amino group on membrane, and results in opening of the base ring [21], which may abolish base-pairing and impose stero-obstacle in formation of a probe–target duplex [15]. This potential problem may be particularly significant for siRNA detection as siRNA is short and the base involving crosslink resides in the same region for hybridization to probe. Thus, the EDC-mediated chemical crosslink [15], which forms a covalent bond between the terminal phosphate group of siRNA and a free amine group on a membrane, can further improve siRNA-detection sensitivity with our probe labeling method. Furthermore, incorporation of locked nucleic acids (LNA), in which the hydrogen atoms of 2′-hydroxy group and 4′-C are replaced with 2′-O,4′-C-methylene group, into the probe sequence significantly increase the melting temperature of duplex formed between LNA-containing DNA and complementary RNA or DNA, thus increasing the sensitivity of LNA probe in Northern blot analysis [22,23,24]. This technique can also be combined into our probe labeling method to improve its sensitivity in both hmwRNA and siRNA detection.

## Figures and Tables

**Table 1:** Comparison between new Northern blot protocol and traditional Northern blot protocol.

Procedure	New Northern Protocol	Traditional mRNA Northern Blot
Step1, Gel preparation		
Gel volume	Low 30–50 mL	High ~200 mL
Time	30 min	30 min
Caution	Pull the comb gently to avoid breaking wells	
Step2, Electrophoresis		
Equipment	Vertical electrophoresis 120 V	Horizontal electrophoresis 100 V
	Compatible with small RNA Northern blot	Not compatible
Time	45 min	2 to 3 h
Step3, Gel blotting		
Equipment	Semidry transfer	Capillary transfer
Time	90 min	12~16 h
Caution	Gel is thin and easy to break, handle with care, Rain-X can be applied to glass plates for easier disassembly	
Step4, Probe-labeling		
Chemistry	T4 DNA pol reaction + biotin-dCTP	Random labeling kit + α-p32-dCTP
Time	75 min	30 min
Advantage	Accessible to all labs	
Disadvantage		Special permit and facility required, material not easy to obtain.
Step5, Hybridization		
Equipment	Hybridization oven	Hybridization oven
Time	Overnight	Overnight (12~16 h)
Disadvantage		Special permit and facility required
Step6, Signal detection		
Equipment	Use the biotin-detection kit and ChemiDoc XRS System	Use a phosphoscreen and scanner
Time	about 1 h	Usually more than 8 h
Overall time frame	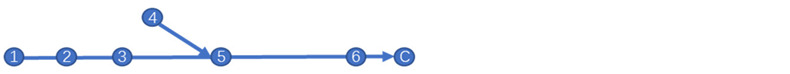	
New protocol		
	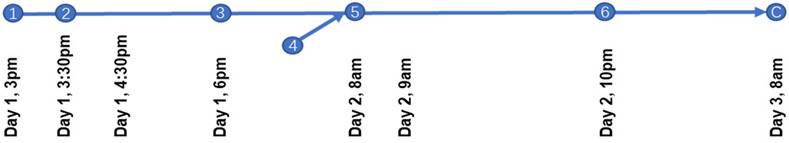	
Old protocol		

## Data Availability

Data is contained within the article or Supplementary Material.

## Supplementary Materials

The following supporting information can be downloaded at: https://www.mdpi.com/article/10.3390/v14122664/s1, Table S1: Sequences of probes used in this study.
